# The complete plastome of *Gymnotheca chinensis* (Saururaceae) and its phylogenetic analysis

**DOI:** 10.1080/23802359.2019.1667908

**Published:** 2019-09-23

**Authors:** Lei Jin, Jin Yang, Changkun Liu, Mengling He, Hanjing Yan

**Affiliations:** a College of Traditional Chinese Medicine, Guangdong Pharmaceutical University, Guangzhou, Guangdong, China;; b Key Laboratory for Plant Diversity and Biogeography of East Asia, Kunming Institute of Botany, Chinese Academy of Sciences, Kunming, Yunnan, China;; c School of Life Science, Yunnan University, Kunming, Yunnan, China;; d Key Laboratory of State Administration of Traditional Chinese Medicine for Production & Development of Cantonese Medicinal Materials, Guangzhou, Guangdong, China

**Keywords:** *Gymnotheca chinensis*, complete plastome, phylogenetic analysis

## Abstract

The complete plastome of *Gymnotheca chinensis*, an important medicinal herb, was firstly elucidated and analyzed in this study. The plastome is 161,621 bp in size, which comprises of one large single-copy (LSC) region and one small single-copy (SSC) region of 89,291 bp and 18,592 bp, respectively, separated by a pair of IR regions of 26,869 bp each. It encodes a total of 132 genes, including 87 protein-coding genes, 37 tRNA, and 8 rRNA. The phylogeny robustly supports that *G. chinensis* is sister to the clade including *Piper kadsura*, *Piper cenocladum*, *Saruma henryi*, *Asarum sieboldii*.

*Gymnotheca chinensis* Decne, which is one of the endemic genera of seed plants in China, is a perennial herb of the family Saururaceae (Xia and Anthony 1999). The whole plants of *G. chinensis* have long been used as a traditional herbal medicine for the treatment of contusions, strains, diarrhea, dysentery, cough and other diseases (Song et al. 2001; Chen et al. 2018). However, most researches were focused on the phytochemical investigation (Xiao et al. 2014, 2016), and no genome data was available. To facilitate its genetic research and the development of conservation value of *G. chinensis*, in the study, we sequenced the complete plastome of *G. chinensis* for the first time.

The healthy and fresh leaves of *G. chinensis* were collected from the Botanical Garden of Kunming Institute of Botany, Chinese Academy of Sciences (25°8′21′′N, 102°44′25′′E). Voucher (Y. Ji 2017085) was identified by Dr. Yunheng Ji, and deposited in the herbarium of Kunming Institute of Botany, Chinese Academy of Science (KUN). Genomic DNA was extracted from 20 mg leaf samples by the modified CTAB method (Yang et al. 2014). Approximately 5 µg of purified genomic DNA was sheared by sonication to generate fragments of 500 bp length for constructing shotgun library. We then sequenced the library using Illumina Hiseq 2000 sequencing platform at BGI (Wuhan, Hubei, China). We assembled the plastome using the method described by Jin et al. (2018) with *Asarum sieboldii* plastome (MG551543) as reference. The assembled genome was annotated in Geneious V10.2 (Kearse et al. 2012), and manually checked for start and stop codons and intron/exon boundaries. The validated complete plastome of *G. chinensis* was deposited in the NCBI GenBank database under the accession number MN263889.

The circular plastome of *G. chinensis* is 161,621 bp in size, which comprises of one large single-copy (LSC) and one small single-copy (SSC) regions of 89,291 bp and 18,592 bp, respectively, separated by a pair of IR regions of 26,869 bp each. It encodes a total of 132 genes, including 87 protein-coding genes, 37 tRNA, and 8 rRNA. Intron-exon structure analysis indicates that 18 genes (12 protein-coding genes and 6 tRNA genes) contain intron, of which, 3 protein-coding genes (*ycf*3, *clp*P and *rps*12) have two introns. The overall G/C content in the *G. chinensis* plastome is 38.30%, and the corresponding value for the IR regions (43.00%) is higher than that of the LSC and SSC regions (36.70% and 32.20%, respectively).

The relationships among Piperales species were reconstructed based on complete plastome DNA sequences, using standard maximum-likelihood (ML) methods (Stamatakis 2014). *Drimys granadensis* was selected as the outgroup. ML analysis was conducted with 1000 bootstraps under the GTRCAT substitution model. The phylogenetic analysis robustly supports that *G. chinensis* is sister to the clade including *Piper kadsura*, *Piper cenocladum*, *Saruma henryi*, and *A. sieboldii* (Figure 1).

**Figure 1.**
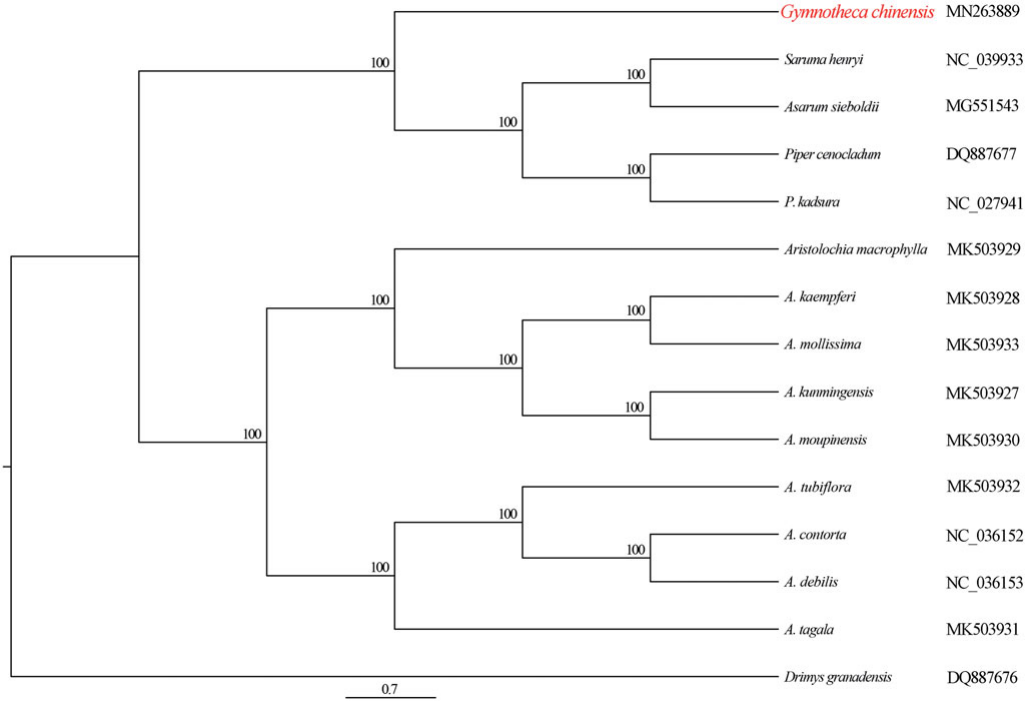
Phylogenetic relationships of *Gymnotheca chinensis* with other Piperales species. The numbers represent bootstrap values.
